# Comparative study of ^18^F-FDG-PET/CT imaging and serum hTERT mRNA quantification in cancer diagnosis

**DOI:** 10.1002/cam4.508

**Published:** 2015-08-15

**Authors:** Bingqiong Ping, Satoshi Tsuno, Xinhui Wang, Yoshitaka Ishihara, Taro Yamashita, Keigo Miura, Fuminori Miyoshi, Yuki Shinohara, Tsutomu Matsuki, Yoshio Tanabe, Noriaki Tanaka, Toshihide Ogawa, Goshi Shiota, Norimasa Miura

**Affiliations:** 1 Division of Pharmacotherapeutics Department of Pathophysiological and Therapeutic ScienceFaculty of Medicine, Tottori University86 Nishicho, Yonago, Tottori, 683-8503, Japan; 2 PEZY-Pharma86 Nishicho, Yonago, Tottori, 683-8503, Japan; 3 Division of Radiology, Department of Pathophysiological and Therapeutic Science, Faculty of Medicine, Tottori University36-1 Nishicho, Yonago, Tottori, 683-8504, Japan; 4 Department of Radiology, Tottori Municipal Hospital1-1 Matoba, Tottori, Tottori, 680-8501, Japan; 5 Division of Molecular and Genetic Medicine, Department of Genetic Medicine and Regenerative Therapeutics, Tottori University School of Medicine86 Nishicho, Yonago, Tottori, 683-8503, Japan

**Keywords:** ^18^F-FDG, cancer, diagnosis, hTERTmRNA, PET/CTPET/CT

## Abstract

We have reported on the clinical usefulness of human telomerase reverse transcriptase (hTERT) mRNA quantification in sera in patients with several cancers. Positron emission tomography–computed tomography (PET/CT) using ^18^F-fluorodeoxyglucose (FDG) has recently become an excellent modality for detecting cancer. We performed a diagnostic comparative study of FDG-PET/CT and hTERT mRNA quantification in patients with cancer. Four hundred seventy subjects, including 125 healthy individuals and 345 outpatients with cancer who had received medical treatments for cancer in their own or other hospitals, were enrolled. The subjects were diagnosed by FDG-PET/CT, and we measured their serum hTERT mRNA levels using real-time RT-PCR, correlating the quantified values with the clinical course. In this prospective study, we statistically assessed the sensitivity and specificity, and their clinical significance. hTERT mRNA and FDG-PET/CT were demonstrated to be correlated with the clinical parameters of metastasis and recurrence (*P* < 0.001), and of recurrence and tumor number in cancer compared with noncancer patients, respectively. A multivariate analysis showed a significant difference in the detection by FDG-PET/CT, ^18^F-FDG uptake, the detection by hTERT mRNA, and age. The use of both FDG-PET/CT and hTERT mRNA resulted in a positivity of 94.4% (221/234) for the detection of viable tumor cells. FDG-PET/CT is superior to hTERT mRNA quantification in the early detection of cancer and combinative use of FDG-PET/CT and hTERT mRNA may improve the diagnostic accuracy of cancer.

## Introduction

Circulating nucleic acids (CNAs) are defined as RNA and DNA that are detected in biological fluids devoid of cellular material 1. Free CNAs in the bloodstream have the potential to serve as biomarkers for certain cancers and disease states 2. Free circulating RNA and exosomal RNA in the plasma and serum are generally present as short fragments ranging from 22 to 1000 nt 3. The exosomes, which are 40- to 100-nm membrane vesicles, contain cell-specific proteins, lipids, and RNAs and can be found in saliva, blood, urine, amniotic fluid, and malignant ascitic fluids, among other biological fluids 4. They are secreted by most cell types and are then transported to other cells 5,6. Recent work has demonstrated the presence of mRNA as well as microRNAs within exosomes, which depends on the tumor cell type from which they are secreted. For this reason, exosomal RNAs may serve as biomarkers for various diseases, including cancer 7–9. We reported previously on the clinical usefulness of quantifying human telomerase reverse transcriptase (hTERT) mRNA in the serum of patients with hepatoma, lung cancer, ovarian cancer, gastric cancer, and esophageal cancer 10–14.

Fluorodeoxyglucose (^18^F-FDG) positron emission tomography/computed tomography (FDG-PET/CT) is an imaging modality that is increasingly being used to assess a patient's cancer stage and response to therapy, as well as for surveillance after treatment for various malignancies 15,16. The diagnostic precision of FDG-PET/CT imaging seems to be improving through analysis of the cause of the incidentally noted FDG uptake in benign tumors 17. Furthermore, the morphological complexity assessed by CT combined with the heterogeneous FDG uptake, which was determined by PET, improved the diagnostic accuracy.

In this study, we performed a comparative study of the rate of cancer detection by two methods, namely, serum hTERT mRNA quantification and FDG-PET/CT, to clarify the efficacy of both modalities for cancer diagnosis.

## Materials and Methods

### Patients and sample collection

**Figure 1:**
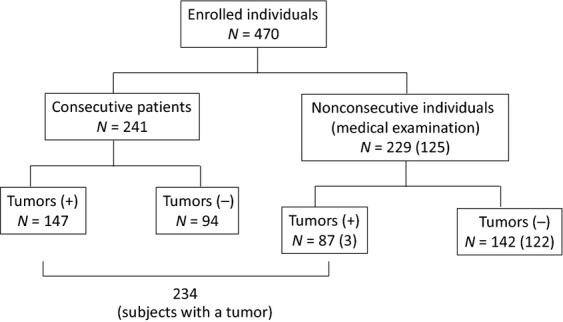
The classification of the subjects enrolled in this study. The study population of 470 individuals was divided into two groups consisting of 241 consecutive patients and 229 nonconsecutive individuals, including 125 healthy individuals for medical examination (see the Materials and Methods section for details).

A total of 470 subjects (including 125 healthy individuals and 345 consecutive patients who were admitted to Tottori University-related hospitals and the Tottori Municipal Hospital between March 2011 and March 2013) were enrolled in this prospective study. All cancer diagnosis including recurrences or metastasis was pathologically and clinically confirmed before or after PET-CT by the physicians in charge in respective hospital that patients had finally visited, according to each clinical practice guideline for cancer diagnosis (Japan Society of Clinical Oncology). Of those individuals, 104 had undergone anticancer therapy more than 1 year previously and were categorized as nonconsecutive patients. Of the consecutive patients, 147 were finally diagnosed as having viable cancer, and 94 individuals were estimated to not have cancer cells in the body. Of the 229 nonconsecutive individuals, including 125 healthy individuals, 87 had a tumor, and the healthy individuals had a medical examination in a cancer screening purpose. Three of the 125 healthy individuals were finally identified as having tumors clinically by medical doctors in charge at each district. A total of 142 individuals were diagnosed as being in a healthy state without cancer (Fig.1). The classification of cancer patients (type of cancer, patients treated before, and total positive patients in hTERT/FDG-PET/CT) are summarized in Table1.

**Table 1:** Profiles and positivity of hTERT mRNA and FDG-PET/CT in 234 cancer patients

	Total number (*N*)	Total positive patient (hTERT/PET/CT)	Patient with therapy (*N*)	Positive patient (hTERT/PET/CT)
Lung cancer	96	54/69	23	10/9
Colon cancer	58	36/35	24	15/14
Malignant lymphoma	38	21/14	19	12/6
Gastric cancer	25	15/13	10	4/4
Pancreatic cancer	17	9/14	4	0/4
Nasopharyngeal carcinoma	17	11/12	3	1/2
Esophageal cancer	12	10/8	2	2/2
Breast cancer	12	6/3	5	3/1
Renal cell carcinoma	7	5/6	3	2/2
Ovarian cancer	7	6/6	7	6/6
Hepatoma	7	5/3	1	1/0
Uterine cancer	4	4/1	1	1/0
Prostate cancer	4	0/0	0	—
Cholangiocarcinoma	4	2/4	1	0/1
Postperitoneal malignancy	4	2/3	1	0/0
Thyroid cancer	3	1/1	1	0/0
Seminoma	3	1/2	0	0/0
Parotid gland cancer	2	1/2	0	0/0
Brain cancer	2	1/1	0	0/0
Tongue cancer	1	1/1	0	0/0
Malignant melanoma	1	0/0	1	0/0
Submandibular gland cancer	1	1/1	0	0/0
Osteosarcoma	1	1/1	0	0/0
Cheilocarcinoma	1	1/0	0	0/0
Gingival cancer	1	1/1	1	1/1
Sebaceous gland sarcoma	1	1/0	0	0/0
Angiosarcoma	1	1/1	0	0/0
Mesentery sarcoma	1	1/1	0	0/0

The 234 cancer patients were diagnosed with 28 different types of cancer and were classified according to the total patient number, total number of positive patients (hTERT mRNA and FDG-PET/CT), number of patients treated with any therapies, and total number of positive patients treated with any therapies (hTERT mRNA and FDG-PET/CT). hTERT, human telomerase reverse transcriptase; FDG, fluorodeoxyglucose; PET, positron emission tomography; CT, computed tomography.

FDG-PET/CT readers were blinded because they did not know the spots obtained from the images were derived from malignant tumors. The clinicopathological characteristics (age, gender, tumor number, tumor size, presence of recurrence, presence of metastasis, status of therapy for malignancies, evaluation time [before or after therapy], and FDG uptake [late − early]) were evaluated. The information on the tumor markers was insufficient for analysis, but 44 patients had a tumor marker specific to their type of cancer, with 25 (56.8%) patients having high hTERT mRNA levels (more than 907 copies was the cut-off value) and 16 (36.4%) patients being diagnosed with cancer by FDG-PET/CT. A total of 234 subjects were diagnosed with cancer.

A total of 122 healthy individuals (including 72 males and 50 females), excluding three subjects who had a tumor, served as the controls. The male and female subjects consisted of 285 and 185 individuals, respectively. Informed consent was obtained from each patient or individual, and the study protocols followed the ethical guidelines of the 1975 Declaration of Helsinki and were approved by the human research committee of Tottori University. The cancer therapies included chemotherapy, radiotherapy, surgical resection, endoscopic treatment, or multidisciplinary therapy. Regarding the follow-up of the patients, blood samples were taken by the physicians in charge. A total of 370 serum samples obtained from the Tottori Municipal Hospital were stored at −80°C and were transferred to Tottori University to be kept at 0°C for 3 h.

### RNA extraction and real-time quantitative RT-PCR

The harvesting of the serum samples was performed as previously described 13. Centrifugation of collected blood and harvesting serum samples were done by using three steps of centrifugation (800*g* with 0.45 Am filtration, 1000*g*, and 1500*g*) to decrease lymphocyte to a minimum. RNA was extracted with a DNase treatment from the serum using SV Total RNA Isolation System (Promega Corp. Madison, WI), as reported previously with modification regarding SYBR Green I and polymerase 12,18. After the elution of RNA in 50 *μ*L of RNase-free water, the quantitative RT-PCR was performed using 2.5 *μ*L of RNA extraction and a KAPA SYBR FAST One-Step qRT-PCR Kit for hTERT (Nippon Genetics Co., Ltd., Tokyo, Japan). The RT-PCR conditions consisted of an initial incubation at 42°C for 5 min, followed by a 5-min inactivation period at 95°C; 40 cycles at 99°C (1 sec), 55°C (10 sec), and 72°C (15 sec); and a 20-sec melting period at 40°C. The dynamic ranges of the real-time PCR analysis of the hTERT mRNA were more than ∼1 copy in this assay. To set up the cut-off value, we compared the data measured in cancer patients with those in healthy individuals and calculated from the output of receiver operating characteristic (ROC) curve analysis (SPSS: Statistical Package for the Social Sciences). OD260/OD280 of RNA used for the quantification ranged from 1.65 to 2.01.

### ^18^F-FDG-PET/CT procedure and interpretation

The ^18^F-FDG-PET/CT (Aquiduo-16; Toshiba, Tokyo, Japan) examinations were carried out using a standard protocol. The patients fasted for at least 6 h before the imaging. The serum glucose levels were measured at the time of the ^18^F-FDG injection and were less than 150 mg/dL in all the patients. A low-dose, noncontrast CT scan was conducted first for the attenuation correction. A transverse emission scan was then initiated from the feet to the head in 7–8 different bed positions ∼60 and 120 min after the administration of 3.7 MBq/kg of ^18^F-FDG. In this estimation, we used FDG uptake [SUV max (late − early)] for improving the detection rate. The attenuation-corrected ^18^F-FDG-PET/CT images were interpreted by the consensus of two or more experienced nuclear medicine specialists who were unaware of the clinical information.

The standardized uptake value (SUV) was assessed in the region of interest (ROI), which was drawn over the areas of maximum intensity in each lesion. The SUVs for FDG were calculated for the ROI using the standard formula. The maximum/minimum SUV max/min value was obtained from the image (60-min image/120-min image) with the highest SUV max/min. A positive malignant ^18^F-FDG uptake, estimated as SUV max − SUV min, was defined as an abnormal increase compared with the background activity in a normal contralateral structure or the surrounding tissues, and vice versa. A final decision was made by consensus based on an evaluation of the results of the ^18^F-FDG-PET/CT and the characteristics of the conventional workup.

### Statistical analysis

The statistical analysis was performed using SPSS 22.0 software (SPSS Corp., Tokyo, Japan). Stratified categories of each clinical parameter were evaluated by a *t*-test, Pearson's correlation coefficient, or a multivariate analysis. To assess the accuracy of the diagnostic tests, the matched data sets regarding FDG-PET/CT and hTERT mRNA and cut-off value were analyzed using ROC curve analysis, based on the definition of accuracy.

## Results

A total of 470 enrolled individuals were divided into two groups consisting of 241 consecutive patients and 229 nonconsecutive individuals. There were 234 subjects with a final clinical diagnosis of a tumor (Fig.1). The examined patients with cancer were categorized according to the cancer type. Lung cancer was the most common type of cancer (96/234, 41%; before therapy: 73/96, after therapy: 23/96), followed by colon cancer (58/234, 24.8%), malignant lymphoma (38/234, 16.2%), gastric cancer (25/234, 10.7%), pancreatic cancer and nasopharyngeal carcinoma (17/234, 7.3%), and esophageal cancer and breast cancer (12/234, 5.1%) (Table1). In patients with lung cancer, pancreatic cancer, or cholangiocarcinoma, FDG-PET/CT was superior to hTERT mRNA with respect to diagnostic accuracy. For the other types of cancers, both modalities were almost equal in accuracy. In patients with malignant lymphoma, breast cancer, hepatoma, or uterine cancer, hTERT mRNA quantification was superior to FDG-PET/CT with respect to diagnostic precision. The PCR yielded products of 143 bp for hTERT. The RT-PCR assay was repeated twice, and the quantification was confirmed using LineGene (TOYOBO, Tokyo, Japan) with reproducibility.

**Figure 2:**
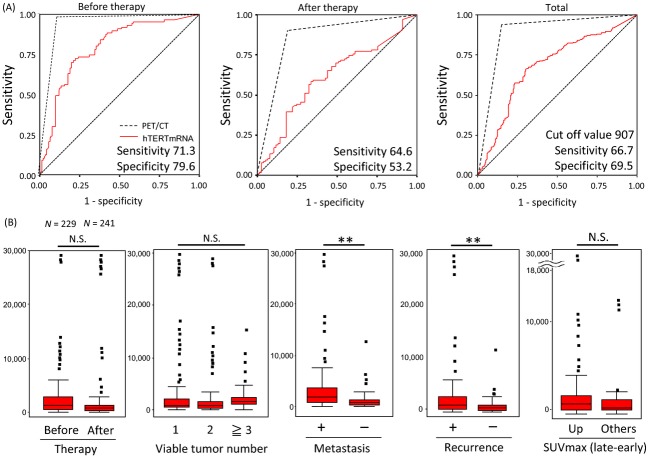
(A) The ROC curve analyses are shown separately according to the results before (left: *N* = 229) and after (middle) therapy. Although the analysis of hTERT mRNA in the total study population resulted in a sensitivity of 66.7% and a specificity of 69.5%, when the data were limited to only the data obtained before therapy, the specificity of hTERT mRNA increased to 79.6%. However, FDG-PET/CT had a sensitivity of more than 90.0% and a specificity of more than 81.0% (Fig. S2). The cut-off value was 907 copies/200 *μ*L. (B) The hTERT mRNA levels were statistically analyzed according to the following variables: therapy status (before or after), tumor number, presence or absence of metastasis, presence or absence of recurrence, and FDG uptake [SUV max (late − early)]. There were significant differences in metastasis and recurrence according to a *t*-test (**P < 0.01 for each). ROC, receiver operating characteristic; hTERT, human telomerase reverse transcriptase; FDG, fluorodeoxyglucose; PET, positron emission tomography; CT, computed tomography; SUV, standardized uptake value.

The diagnostic accuracy of FDG-PET/CT and hTERT mRNA quantification in cases of malignant tumors was estimated after stratification for the presence or absence of a malignant tumor, resulting in positive predictive values of FDG-PET/CT and hTERT mRNA quantification of 84.1% and 66.7% and negative predictive values of 95.3% and 67.8%, respectively. The diagnostic accuracy of both modalities (FDG-PET/CT and hTERT mRNA quantification) in all the individuals was 94.4% (221/234) (Fig. S1). When the data were categorized according to therapeutic treatment status (i.e., before or after), the ROC curve analysis showed that the sensitivity and specificity of hTERT mRNA quantification for cancer detection were 71.3% and 79.6% (cut-off value was 907 copies/200 *μ*L of serum), respectively (Fig.2A: left), suggesting that hTERT mRNA quantification is more useful for the early detection of malignancies compared with disease evaluation during follow-up (Fig.2A: middle, after therapy; right, overall estimation). The information on the tumor markers was insufficient for analysis, but 44 patients had a tumor marker specific to their type of cancer, with 25 (56.8%) patients having high hTERT mRNA levels and 16 (36.4%) patients being detected with cancer by FDG-PET/CT.

The sensitivity, specificity, area under cover (AUC), SE, *P* value, and 95% CI of hTERT mRNA quantification (top) and FDG-PET/CT (bottom) in the patients are shown in Figure S2. Although the hTERT mRNA quantification method revealed significant differences in the presence of metastasis or recurrence (*P* < 0.01 by a *t*-test) (Fig.2B), it did not show any significant differences in tumor number or FDG uptake [SUV max (late − early)] before or after therapy. FDG-PET/CT revealed significant differences in tumor number and the presence of recurrence (*P* < 0.01 by a *t*-test). The SUV max (late − early) (plus: 144 cases, minus: 22 cases) corresponding to increased FDG uptake showed significant differences in the presence of metastasis (*P* < 0.01 by a *t*-test).

**Figure 3:**
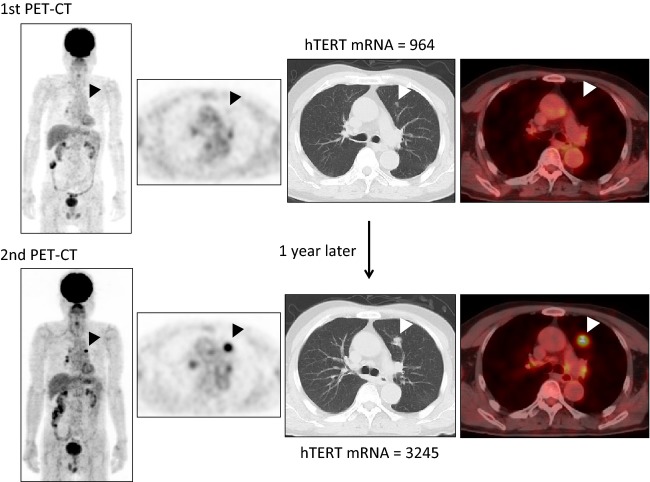
A representative case of a patient with lung cancer. The diagnosis was confirmed by needle aspiration cytology. The top and bottom columns include four images (PET-CT, axial view of PET [MIP: image], axial view of CT, and fusion image [selected transaxial fused PET/CT slice] from the right to left); the bottom images were acquired a year after the top images to enable a more precise diagnosis. The fusion image was used for enhancing FDG accumulation on selected slice. The first PET-CT study did not diagnose the findings (shown by an arrowhead) as indicative of malignancy yet. The hTERT mRNA increased from 964 to 3245 copies in the 1-year interval between imaging studies, with both values indicating positivity. The bottom images demonstrate the increased size of a lung tumor over time, suggesting the potential use of hTERT mRNA quantification as a complementary diagnostic technique. PET, positron emission tomography; CT, computed tomography; MIP, maximum intensity projection; FDG, fluorodeoxyglucose; hTERT, human telomerase reverse transcriptase.

Pearson's correlation test showed that hTERT mRNA levels had a significant correlation with the presence of metastasis and recurrence, while FDG-PET/CT imaging had a significant correlation with tumor number, the presence of metastasis, and recurrence (Table2A). The multivariate analysis (using data weighted by tumor presence) revealed significant differences in FDG-PET/CT (*P* < 0.001), FDG uptake (*P* < 0.01), and hTERT mRNA results (*P* < 0.01) (Table2B). Significant correlations between hTERT mRNA upregulation and detection by FDG-PET/CT were confirmed in a representative patient with lung cancer, as shown in Figure3.

**Table 2:** (A) According to a Pearson's correlation test, hTERT mRNA levels showed significant differences with respect to metastasis and recurrence, while FDG-PET/CT imaging showed significant differences with respect to metastasis, recurrence, and tumor number (*P* < 0.01 for each). SUV max (late − early) showed significant differences with respect to metastasis (*P* < 0.01). (B) The multivariate analysis (using data weighted according to tumor presence) showed significant differences in hTERT mRNA quantification, the diagnosis using FDG-PET/CT, and the presence of FDG uptake results (*P* < 0.01 for each)

(A) Pearson's correlation test		
Diagnostic factor	Tumor-related factor	*P* value
hTERTmRNA	Metastasis	0.042
	Recurrence	0.037
PET/CT	Tumor number	<0.01
	Metastasis	<0.01
	Recurrence	<0.01
FDG uptake [SUV max (late − early)]	Metastasis	<0.01

(B) Multivariate analysis (using data weighed with the presence of tumor)	
Diagnostic factor	*P* value
hTERTmRNA	0.005
PET/CT	<0.001
Increasing FDG uptake	0.003

hTERT, human telomerase reverse transcriptase; FDG, fluorodeoxyglucose; PET, positron emission tomography; CT, computed tomography; SUV, standardized uptake value.

## Discussion

The possible roles of RNA in monitoring treatment and providing a personalized clinical diagnosis and prognosis in oncology have been previously discussed. There have been conflicting data regarding the stability of RNA in peripheral blood, with Glyceraldehyde 3-phosphate dehydrogenase (GAPDH) mRNA apparently being stable for 24 h at room temperature 19. However, *β*-actin mRNA decreased 10-fold in 2 h and nearly 100-fold in 5 h, most likely due to RNase activity 20. As there was a significant increase in the RNase levels of cancer patients, circulating RNA molecules should be protected to enable their use in cancer diagnostics 21. In fact, because the RNA molecules encapsulated within exosomes are protected from degradation by RNases, they can be efficiently recovered from biological fluids, such as plasma or serum 22–24.

Evidence has recently been accumulating that exosomes act as cellular messengers, conveying information to distant cells and tissues within the body 25–30. Even if the RNA molecules in the blood were promptly treated to achieve purification, the nucleolytic degradation that occurs during the transportation of the blood, serum, or RNA for clinical use should be prevented. We performed the serum separation within 2 h in this study, and the serum was stored at −80°C. However, it took 3 h to transport the serum from the collaborative hospital for the hTERT mRNA measurement at −20°C. Thus, RNA degradation could not be avoided to some extent because an innovative technique for RNA stabilization in the process of purification from human fluids has not yet been satisfactorily developed.

The hybrid imaging modality of PET/CT allows for the simultaneous assessment of molecular and morphological information. FDG-PET/CT represents an efficient imaging modality for whole body staging and restaging. The glucose analog FDG is the most widely used PET and PET/CT radiopharmaceutical in clinical oncology protocols. FDG-PET and PET/CT have been used to stage and restage tumor patients in numerous studies. Currently, PET/CT and PET/MR are excellent imaging modalities for detecting cancerous lesions and have been increasingly utilized. FDG-PET/CT generally allows for an assessment of the site and extent of the recurring disease with an accuracy of ∼83% 31,32.

The primary uses of FDG-PET and PET/CT in oncology are for the diagnosis and detection of lung cancer, esophageal cancer, colorectal cancer, head and neck cancer, lymphoma, and breast cancer (among other tumor types). Many reviews have been conducted on the primary diagnosis, staging, and diagnosis of recurrent disease (local disease, lymph node metastases, and distant metastases) 33. Additionally, the SUV and other measurements of tumor uptake of FDG on PET can potentially be supplemented with additional imaging parameters derived from either PET images or the CT component of the integrated PET/CT examinations, including tumor size, CT attenuation, texture (reflecting tumor heterogeneity), and blood flow 34.

In this study, we performed a comparative investigation of FDG-PET/CT imaging and an hTERT mRNA quantification technique that we developed to detect evidence of cancer cells circulating in the blood under cell-free conditions. In a previous investigation, a comparative study of serum markers and PET/CT was performed 35. In this study, we modified the real-time one-step RT-PCR technique to improve the detection sensitivity by changing the fluorescent dye; this modification hardly inhibited the PCR, suggesting that FDG-PET/CT imaging is a better modality than hTERT mRNA quantification for cancer diagnosis. Because we assumed that these significant differences in tumor number and the presence of recurrence are enhanced by differences between the 60 min and delayed FDG-PET/CT images, we confirmed that SUV max (late − early) was also an excellent modality to evaluate a clinical status in patients with cancer and a very useful parameter for detecting viable cancer cells; however, hTERT mRNA is applicable for detecting cancer cells in a primary examination, although the nucleolytic degradation of mRNA cannot be prevented during the transportation of serum samples 36. We reported that hTERT mRNA measurements had more than an 80% positivity rate for detecting lung cancer, hepatoma, and ovarian cancer.

In this study, the detection rates of lung cancer and hepatoma were 56% and 71%, respectively, while previous studies yielded detection rates of 72.7% and 88.2%, respectively 10,11. Some patients had a higher (more than 2×) level of hTERT mRNA, irrespective of noncancerous conditions. In contrast, a lower or nonexistent hTERT mRNA level was observed in patients with lung cancer nodules. Thus, although the reasons for the mutually exclusive results regarding the hTERT mRNA levels remain unknown, we think that hTERT mRNA causes RNA degradation in the process of treating the samples and that this technique should therefore be supported by other complementary markers or modalities. The combined positive and negative FDG-PET/CT and hTERT mRNA quantification detection rates were 94.4% and 5.6%, respectively (Fig. S3). We are expecting the discovery of an innovative material, container, or apparatus with which to stabilize RNA molecules or exosomes to facilitate RNA protection 37–39. Additionally, the imaging modality used in this study will be more applicable for clinical medicine than before, by the estimation of delayed phase.

## Supporting Information

**Figure S1.** The diagnostic characteristics of FDG-PET/CT and hTERT mRNA quantification.

**Figure S2.** The usefulness of both diagnostic modalities for cancer detection.

**Figure S3.** Diagnostic accuracy of each modality.
