# Supplementary material for: Comparative study of 18F-FDG-PET/CT imaging and serum hTERT mRNA quantification in cancer diagnosis
Source: Cancer Med. 2015 Aug 15;4(10):1603–11. doi: 10.1002/cam4.508 (PMC4618631; doi:10.1002/cam4.508)
Supplement: Supplementary file 1 [file cam40004-1603-sd1.pdf]

Supplementary File 1

		presence of tumor		
		+	−	total
detection by PET/CT	+	197 (84.1%/94.7%)	11 (4.7%/5.3%)	208
	−	37 (15.9%/14.1%)	225 (95.3%/85.9%)	262
	total	234	236	470

		presence of tumor		
		+	−	total
detection by hTERTmRNA	+	156 (66.7%/68.4%)	72 (30.5%/31.6%)	228
	−	78 (33.3%/32.2%)	164 (69.5%%/67.8%)	242
	total	234	236	470

Title: The diagnostic characteristics of FDG-PET/CT and hTERT mRNA quantification

The diagnostic characteristics of FDG-PET/CT and hTERT mRNA quantification are shown. The PPV and NPV of FDG-PET/CT were 84.1 and 95.3%, respectively. The detection rate associated with FDG-PET/CT was 89.8%. The PPV and NPV of hTERT mRNA quantification were 66.7 and 69.5%, respectively. The detection rate associated with hTERT mRNA quantification was 68.1%.
