# Supplementary material for: Comparative study of 18F-FDG-PET/CT imaging and serum hTERT mRNA quantification in cancer diagnosis
Source: Cancer Med. 2015 Aug 15;4(10):1603–11. doi: 10.1002/cam4.508 (PMC4618631; doi:10.1002/cam4.508)
Supplement: Supplementary file 2 [file cam40004-1603-sd2.pdf]

Title: The usefulness of both diagnostic modalities for cancer detection Supplementary file 2

before therapy: N = 229 | total: N = 470

(a) hTERTmRNA

AUC	S.E.	P value	95% CI lower limit	95% CI upper limit	AUC	S.E.	P value	95% CI lower limit	95% CI upper limit
.793	.034	.002	.702	.875	.681	.035	.002	.653	.724
Sensitivity 71.3					Sensitivity 66.7				
Specificity 79.6					Specificity 69.5				

(b) PET/CT

AUC	S.E.	P value	95% CI lower limit	95% CI upper limit	AUC	S.E.	P value	95% CI lower limit	95% CI upper limit
.860	.032	.001	.702	.875	.898	.032	.001	.855	.933
Sensitivity 90.4					Sensitivity 95.3				
Specificity 81.6					Specificity 84.2				

To assess the usefulness of the two diagnostic modalities for the detection of cancer, the detection rates in subjects (left column: before therapy; right column: total study population) were calculated using ROC curve analysis. The AUC, S.E., P value, and 95% CI (lower limit and upper limit) for (a) hTERT mRNA quantification and (b) FDG-PET/CT are shown in the patients before therapy (left) and total patient population (right). In addition, the sensitivity and specificity are described for each.
