# Supplementary material for: Comparative study of 18F-FDG-PET/CT imaging and serum hTERT mRNA quantification in cancer diagnosis
Source: Cancer Med. 2015 Aug 15;4(10):1603–11. doi: 10.1002/cam4.508 (PMC4618631; doi:10.1002/cam4.508)
Supplement: Supplementary file 3 [file cam40004-1603-sd3.pdf]

Title: Diagnostic accuracy of each modality

Supplementary file 3

Diagnostic accuracy

modality	detection rate of tumor positivity / negativity
hTERTmRNA	156 / 78<br>(66.7% / 33.3%)
PET/CT	197 / 37<br>(84.2% / 15.8%)
hTERTmRNA/PET/CT	221/ 13<br>(94.4% / 5.6%)
total	234

Legend: The combined use of hTERT mRNA quantification and FDG-PET/CT demonstrated an improved diagnostic accuracy for the detection of the presence of a tumor of 94.4% (221/234).
